# Monolacunary Wells-Dawson Polyoxometalate as a Novel Contrast Agent for Computed Tomography: A Comprehensive Study on In Vivo Toxicity and Biodistribution

**DOI:** 10.3390/ijms25052569

**Published:** 2024-02-22

**Authors:** Marko Stojanović, Mirjana B. Čolović, Jovana Lalatović, Aleksandra Milosavljević, Nada D. Savić, Kilian Declerck, Branimir Radosavljević, Mila Ćetković, Tamara Kravić-Stevović, Tatjana N. Parac-Vogt, Danijela Krstić

**Affiliations:** 1 Department of Pharmacology, Clinical Pharmacology and Toxicology, Faculty of Medicine, University of Belgrade, 11000 Belgrade, Serbia; marko.stojanovic@med.bg.ac.rs; 2 “Vinča” Institute of Nuclear Sciences—National Institute of the Republic of Serbia, University of Belgrade, 11351 Belgrade, Serbia; colovicm@vin.bg.ac.rs; 3 Department of Radiology, University Hospital Medical Center Bežanijska Kosa, 11080 Belgrade, Serbia; lalatovic.jovana@bkosa.edu.rs; 4 Institute of Histology and Embryology, Faculty of Medicine, University of Belgrade, 11000 Belgrade, Serbia; aleksandra.milosavljevic@med.bg.ac.rs (A.M.); mila.cetkovic-milisavljevic@med.bg.ac.rs (M.Ć.); tamara.kravic-stevovic@med.bg.ac.rs (T.K.-S.); 5 Department of Chemistry, KU Leuven, Celestijnenlaan 200F, 3001 Leuven, Belgium; nada.savic@kuleuven.be (N.D.S.); kilian.declerck@kuleuven.be (K.D.); 6 Institute of Medical Chemistry, Faculty of Medicine, University of Belgrade, 11000 Belgrade, Serbia; branimir.radosavljevic@med.bg.ac.rs

**Keywords:** blood gas analysis, CO-oximetry status, biochemical parameters, histological analysis, in vitro computed tomography imaging, in vivo toxicity, monolacunary Wells-Dawson polyoxotungstate, tissue distribution, X-ray attenuation

## Abstract

Polyoxotungstate nanoclusters have recently emerged as promising contrast agents for computed tomography (CT). In order to evaluate their clinical potential, in this study, we evaluated the in vitro CT imaging properties, potential toxic effects in vivo, and tissue distribution of monolacunary Wells–Dawson polyoxometalate, α_2_-K_10_P_2_W_17_O_61_^.^20H_2_O (mono-WD POM). Mono-WD POM showed superior X-ray attenuation compared to other tungsten-containing nanoclusters (its parent WD-POM and Keggin POM) and the standard iodine-based contrast agent (iohexol). The calculated X-ray attenuation linear slope for mono-WD POM was significantly higher compared to parent WD-POM, Keggin POM, and iohexol (5.97 ± 0.14 vs. 4.84 ± 0.05, 4.55 ± 0.16, and 4.30 ± 0.09, respectively). Acute oral (maximum-administered dose (MAD) = 960 mg/kg) and intravenous administration (1/10, 1/5, and 1/3 MAD) of mono-WD POM did not induce unexpected changes in rats’ general habits or mortality. Results of blood gas analysis, CO-oximetry status, and the levels of electrolytes, glucose, lactate, creatinine, and BUN demonstrated a dose-dependent tendency 14 days after intravenous administration of mono-WD POM. The most significant differences compared to the control were observed for 1/3 MAD, being approximately seventy times higher than the typically used dose (0.015 mmol W/kg) of tungsten-based contrast agents. The highest tungsten deposition was found in the kidney (1/3 MAD—0.67 ± 0.12; 1/5 MAD—0.59 ± 0.07; 1/10 MAD—0.54 ± 0.05), which corresponded to detected morphological irregularities, electrolyte imbalance, and increased BUN levels.

## 1. Introduction

Computed Tomography (CT) has been employed as a pivotal imaging technique in both clinical and research domains, providing 3D images with high spatial and temporal resolution. The constant evolution of CT technology, characterized by innovations in image reconstruction algorithms and reduced radiation doses, is largely able to address its limitations. In the field of diagnostic medicine, this non-invasive technique, combined with contrast media, provides detailed 3-dimensional anatomical information of blood vessels, internal organs, and pathological lesions [1,2,3].

However, compared to other clinical imaging techniques such as Magnetic Resonance Imaging, the lack of sensitivity to contrast agents is the main limitation of CT. Furthermore, the conventionally used intravenous contrast agents for CT, iodinated small molecules (derivatives of a 2,4,6-triiodinated benzene ring), have several drawbacks, including adverse reactions [4,5,6] and a heightened risk for patients with specific conditions [7]. In addition, these contrast agents are not specific for any pathology and have limited blood circulation time. Thus, in the realm of medical imaging, the quest for innovative CT contrast agents to enhance diagnostic accuracy and imaging capabilities remains an ongoing endeavor. Recent studies highlight the potential of metallic nanoparticles and polyoxometalate (POM) nanoclusters as promising candidates for developing new-generation CT contrast agents [8,9,10,11,12]. These metal-based compounds possess extended circulation times and favorable X-ray attenuation, whereas their shape, composition, size, and surface characteristics are tunable for desired biomedical applications [13,14]. 

**Scheme 1:**
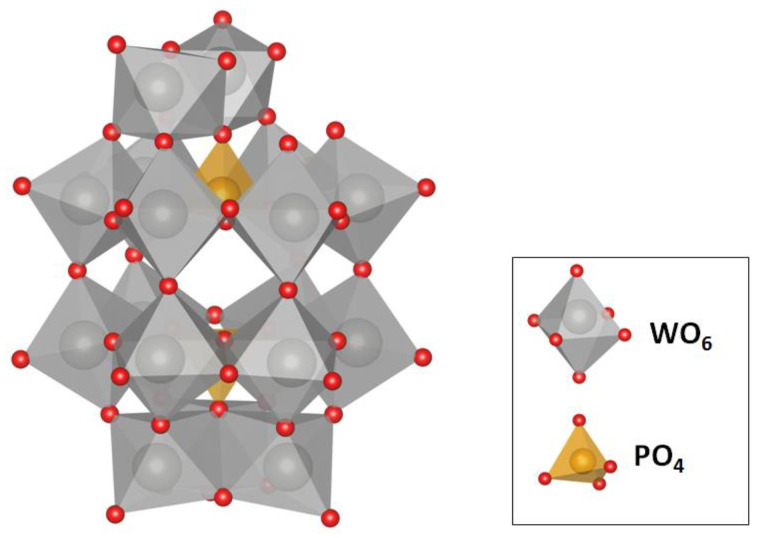
Structure of monolacunary Wells–Dawson polyoxotungstate (mono-WD POM), α_2_-K_10_P_2_W_17_O_61_^.^20H_2_O (red balls–oxygen).

In this study, in vitro CT imaging properties and potential toxic effects of monolacunary Wells–Dawson POM, α_2_-K_10_P_2_W_17_O_61_^.^20H_2_O (mono-WD POM), after intravenous administration in rats were evaluated to achieve a careful balance between imaging performance and potential side effects in the development of new-generation POM-based contrast agents. In addition, ex vivo biodistribution of mono-WD POM was evaluated two weeks after treatment with 1/10, 1/5, and 1/3 of the maximum-administered dose (MAD) by measuring tungsten concentrations in different tissue samples, using Inductively Coupled Plasma-Optical Emission Spectroscopy (ICP-OES). Mono-WD POM (Scheme 1) is a derivative of parent WD-POM (α-/β-K_6_P_2_W_18_O_62_), which was studied as a potential CT contrast in our previous research [9]. Parent WD-POM is partially converted into mono-WD POM at physiological pH by spontaneous removal of one -WO unit [12]. Moreover, de Bournonville et al. reported mono-WD POM as the most suitable non-destructive staining agent for contrast-enhanced microfocus CT of bone and kidney [12]. Generally, the results of this study contribute novel insights into the relationship between structure and toxic properties of POMs, which have been widely explored as promising diagnostic tools and therapeutics [10,11,12,15,16,17]. 

## 2. Results

### 2.1. In Vitro CT Performances

**Figure 1:**
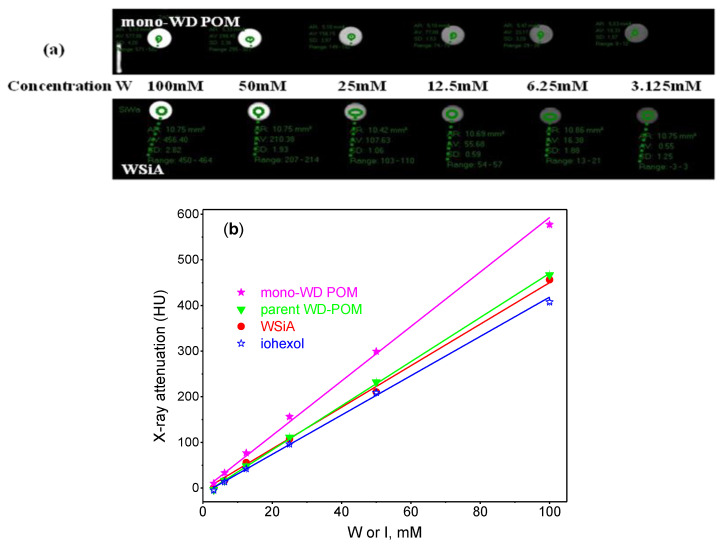
(**a**) Images generated from a clinical CT scanner phantom scan of mono-WD POM and WSiA, ranging from 3.125 to 100 mM W; (**b**) the linear dependence of X-ray attenuation (expressed in Hounsfield units (HU)) on W/I concentration for the tungsten-containing compounds of various structures: Keggine structure POM (12-tungstosilicic acid, WSiA) (red circle), parent WD-POM (green triangle) [9] and its monolacunary derivative (mono-WD POM) (pink solid asterisk), and iodine-containing omnipaque (blue open asterisk) [9].

X-ray attenuation (X-ray source voltage of 80 kV) was measured in vitro in the presence of increasing concentrations of tungsten (3.125–100 mM), one Keggin structure POM (12-tungstosilicic acid, WSiA), and two analogous WD POMs: parent WD [9] and its monolacunary derivative, mono-WD POM (Scheme 1). The corresponding in vitro CT phantom images of mono-WD and WSiA are presented in Figure 1a. In order to compare the contrast performances of these tungsten-containing compounds with a commercially available CT contrast agent that has been employed in clinical practice, X-ray attenuation was also presented for the same iodine concentrations of iodine-based iohexol solution [9]. The results are expressed in Hounsfield units (HU) and presented as a function of W/I concentration in Figure 1b. The obtained experimental results for all tested compounds showed an excellent fit to a linear function (R^2^ > 0.99). The highest HU values for particular concentrations were obtained for mono-WD POM compared to those for both its parent WD compound and WSiA, a POM with Keggin structure. Accordingly, the calculated value of the slope for mono-WD POM was significantly higher compared to parent WD and WSiA (5.97 ± 0.14 vs. 4.84 ± 0.05 and 4.55 ± 0.16, respectively). The obtained values of X-ray attenuation for all studied concentrations were evidently higher for all three tungsten-containing POMs in comparison with the standard iodine-based CT contrast agent, iohexol, for which the slope of the linear function had a value of 4.30 ± 0.09 (Figure 1b).

### 2.2. General Habit of Animals

After the acute oral application of MAD (960 mg/kg mono-WD POM), any unexpected changes in the appearance of skin and visible mucosa, sleeping, feeding, and body weight, as well as mortality, were not observed in the treated Wistar rats. This indicates that the orally applied MAD does not produce severe toxic effects or death in animals.

During a 14-day follow-up period after the intravenous administration of mono-WD POM, a mortality rate was investigated for four experimental groups (five rats per one group): (1) control (saline), (2) 1/3 MAD (320 mg/kg), (3) 1/5 MAD (192 mg/kg), and (4) 1/10 MAD (96 mg/kg), and was 0% in all cases. Indeed, all observed experimental rats (both the treated and control groups) survived (a 100% survival rate) 14 days post-mono-WD POM application intravenously. Furthermore, during the 14 days after the intravenous application of saline/mono-WD POM, the rats were observed in the morning and evening [18,19]. No significant changes in behavior (sleeping and feeding) and body weight (not following the rat age) were detected throughout the follow-up period. The inspection of skin and visible mucosa did not reveal any suspected changes in appearance in all experimental groups (both control and POM-administered animals). 

### 2.3. Laboratory Blood Analysis

On the 14th day post-treatment, arterial blood gas analysis, CO-oxymetry status, electrolyte levels, glucose level, lactate level, creatine level, and BUN values were measured from arterial blood.

#### 2.3.1. Arterial Blood Gas Analysis

Arterial blood gas analysis (Table 1) demonstrates low pH values that were under the normal range for all groups, both control and three treated groups, without statistical differences between them (*p* = 0.0545). pCO_2_ levels were above the upper limit of the normal range for all treated groups. A statistically significant difference was noted only between the control and 1/3 MAD group (*p* = 0.0007). HCO_3_^−^ levels detected in the treated groups (1/5 and 1/3 MAD) were above the upper limit of the normal range. A statistically significant difference in HCO_3_^−^ level was detected between all treated groups and the control group (*p* < 0.0001). The base excess of blood values was significantly higher in the treated groups (*p* = 0.0008) relative to control. The detected control group values were below the lower limit of the normal range. The anion gap values were below the lower limit of the normal range in the 1/3 MAD group, whereas these values were within the normal range for the other groups. A statistically significant difference was obtained between the control and 1/3 MAD, as well as the 1/5 MAD group (*p* < 0.0001).

#### 2.3.2. Arterial Blood CO-Oxymetry Status

The results of CO-oximetry analysis for the control and all three treated groups are shown in Table 2. It was found that the values of total hemoglobin, carboxyhemoglobin, and the oxygen capacity of hemoglobin were within the normal range for all studied groups, and there was no statistically significant difference among groups regarding these parameters (*p* > 0.05). The oxyhemoglobin values were below the lower limit of the normal range in the treated groups. These values were statistically significantly lower in the treated groups compared to the control group (*p* = 0.0001). Obtained methemoglobin values were in the normal range in both control and treated groups. However, values for the treated groups were significantly lower (*p* = 0.0008) relative to the control values. Then, the values of deoxyhemoglobin in the treated groups were highly above the upper limit of the normal range and were statistically significantly higher than the values measured in the control group (*p* < 0.0001). The oxygen saturation was below the normal range in the treated groups, and these values were statistically significantly lower in the 1/5 MAD and 1/10 MAD groups when compared to the control group (*p* = 0.004). Furthermore, the oxygen content of hemoglobin was below the normal range in all treated groups, whereas a statistically significant difference was observed when the control and 1/5 MAD group were compared (*p* = 0.0202).

#### 2.3.3. Arterial Blood Electrolyte Concentrations

The obtained blood values of different electrolytes (Na^+^, K^+^, Cl^−^, Ca^2+^, Mg^2+^, Ca^2+^ normalized to pH 7.4 and Mg^2+^ normalized to pH 7.4) are presented in Table 3. When comparing the values of Na^+^, K^+^, and Cl^−^ between the control and treated groups, there is no statistically significant difference, nor do the obtained values deviate significantly from the normal range. Ca^2+^ values were within the normal range in all groups except for the 1/3 MAD group, whose values were above the upper limit of the normal range. Statistically significantly higher values were detected in the 1/3 MAD group compared to the control group (*p* = 0.0058). The Mg^2+^ values were above the upper limit in all three treated groups, whereas a statistically significant difference was detected between the obtained values for the control and 1/3 MAD group (*p* = 0.0001). The values of Ca^2+^ normalized to pH 7.4 were in the normal range in both control and treated groups, and a statistically significant difference was observed only when the control group was compared to the 1/5 MAD group (*p* = 0.0151). Finally, the values of Mg^2+^ normalized to pH 7.4 were above the upper limit for all three treated groups. A statistically significant difference was obtained between the control group and 1/3 MAD group, as well as the 1/5 MAD group (*p* = 0.002). 

#### 2.3.4. Arterial Blood Glucose Level

**Figure 2:**
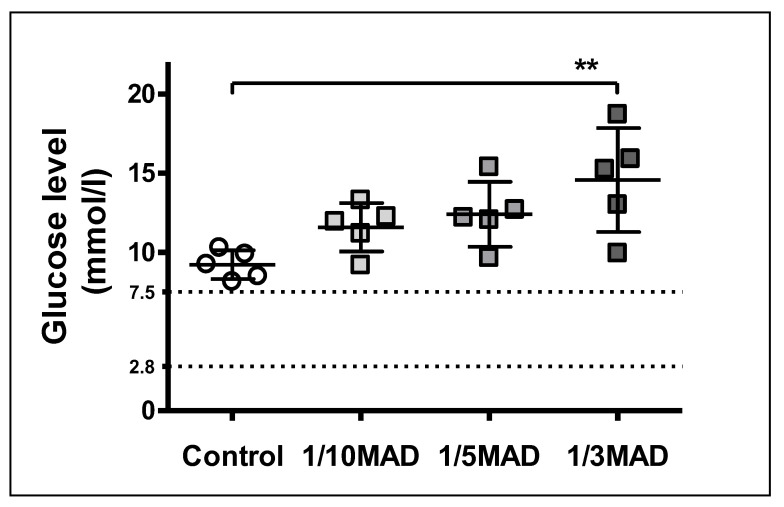
Arterial blood glucose levels in the control group and animals treated with 1/10 MAD, 1/5 MAD, and 1/3 MAD of mono-WD POM. The results are expressed as mean value ± SD. The significant values: ** *p* < 0.01.

The glucose levels (Figure 2) in the control group (9.22 ± 0.91 mmol/L), 1/10 MAD group (11.58 ± 1.53 mmol/L), 1/5 MAD group (12.40 ± 2.05 mmol/L), and 1/3 MAD group (14.56 ± 3.28 mmol/L) were above the upper limit of the normal range (2.8–7.5 mmol/L). A statistically significant difference in glucose level was detected only between the control and 1/3 MAD groups (*p* = 0.0095).

#### 2.3.5. Arterial Blood Lactate Level

**Figure 3:**
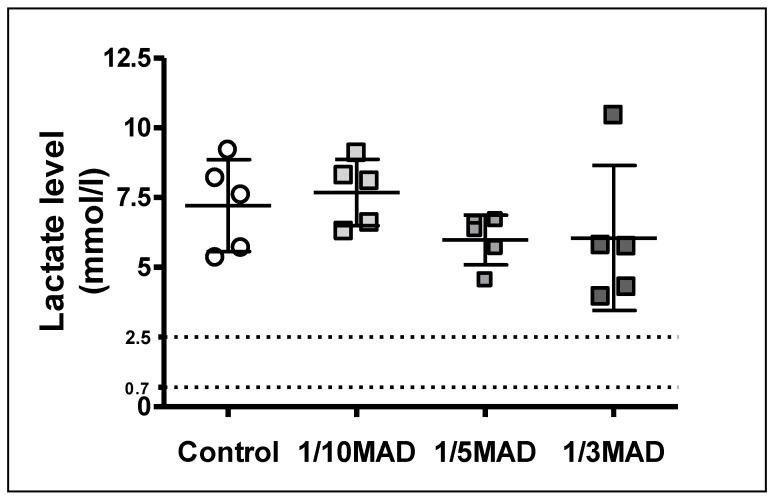
Arterial blood lactate levels in the control group and animals treated with 1/10 MAD, 1/5 MAD, and 1/3 MAD of mono-WD POM. The results are expressed as mean value ± SD.

Lactic acid levels (Figure 3) were above the upper limit of the normal range (0.7–2.5 mmol/L) in the control group (7.21 ± 1.65 mmol/L), 1/10 MAD group (7.68 ± 1.19 mmol/L)), 1/5 MAD group (5.98 ± 0.89 mmol/L), and 1/3 MAD group (6.05 ± 2.60 mmol/L). No statistically significant differences were detected between the control and all treated groups.

#### 2.3.6. Arterial Blood Creatinine Concentrations

**Figure 4:**
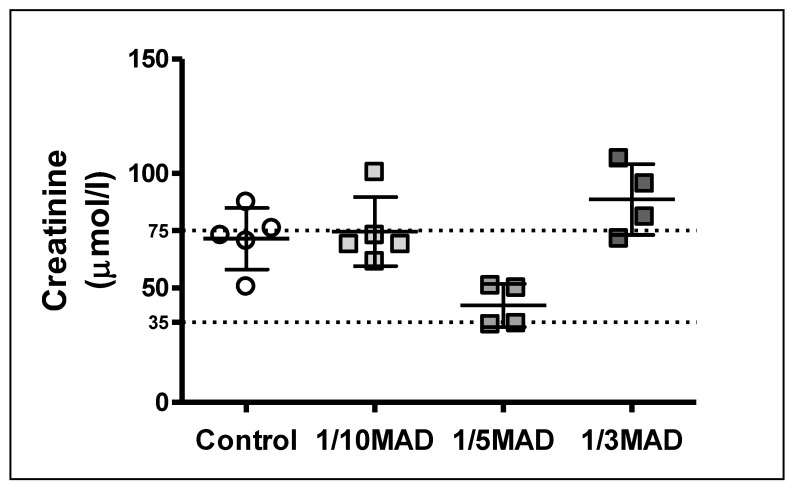
Arterial blood creatinine concentrations in the control group and animals treated with 1/10 MAD, 1/5 MAD, and 1/3 MAD of mono-WD POM. The results are expressed as mean value ± SD.

The creatinine values measured in arterial blood (Figure 4) were within the limits of the normal range (35–75 µmol/L) in the control (71.50 ± 13.42 µmol/L) and 1/5 MAD group (42.38 ± 9.39 µmol/L), just below the upper limit of the normal range in the 1/10 MAD group (74.60 ± 15.06 µmol/L), and above the upper limit of the normal range in the 1/3 MAD group (88.62 ± 14.38 µmol/L), creating a U-shape curve. There were no statistically significant differences between the control group and any of the treated groups (*p* = 0.0507).

#### 2.3.7. Arterial Blood BUN Concentrations

**Figure 5:**
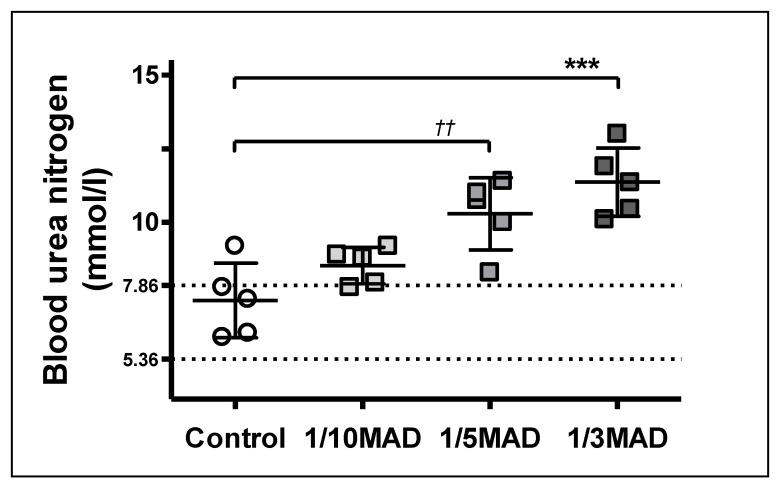
Arterial blood urea nitrogen (BUN) concentrations in the control group and animals treated with 1/10 MAD, 1/5 MAD, and 1/3 MAD of mono-WD POM. The results are expressed as mean value ± S.D. The significant values: *** *p* < 0.001, †† *p* < 0.01.

In the control group, the detected BUN values (7.35 ± 1.27 µmol/L) were within the normal range (5.36–7.86 µmol/L). However, BUN values (µmol/L) were above the upper limit of the normal range in the 1/10 MAD (8.53 ± 0.62), 1/5 MAD (10.39 ± 1.22), and 1/3 MAD (11.36 ± 1.16) groups (Figure 5). A statistically significant difference was obtained between the control group and the 1/5 MAD as well as the 1/3 MAD groups (*p* = 0.0001). 

### 2.4. Histological Analysis

#### 2.4.1. Histopathological Evaluation of Mono-WD POM—Induced Renal Toxicity

**Figure 6:**
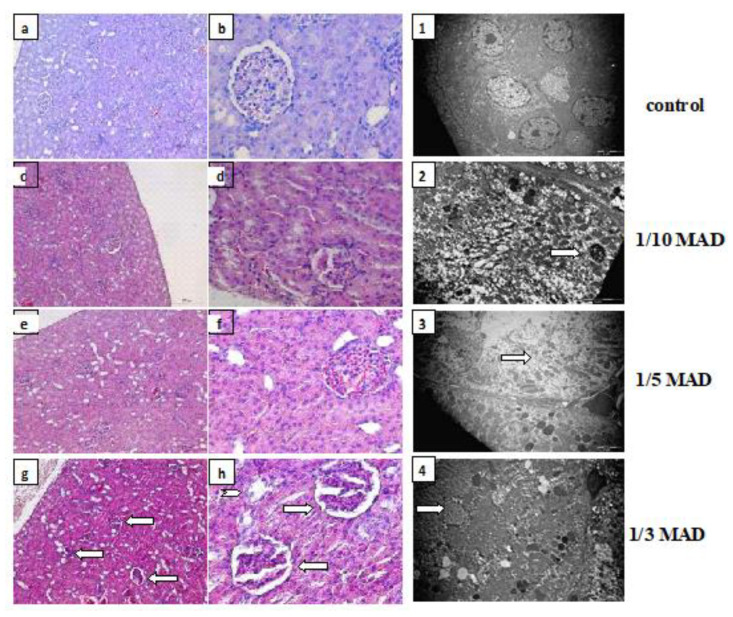
Histopathological evaluation of mono-WD POM-induced renal toxicity. Photographs of HE-stained rat kidney sections (**a**–**h**); image was captured under a light microscope with ×10 (**a**,**c**,**e**,**g**) and ×40 (**b**,**d**,**f**,**h**) magnifications. No differences were observed between the kidney tissues from the control group (**a**,**b**) and rats treated with 1/10 MAD (**c**,**d**) and 1/5 MAD (**e**,**f**), whereas tissue from rats treated with 1/3 MAD (**g**,**h**) showed necrosis of tubular cells (arrowhead) and glomerular sclerosis (arrows). TEM micrographs of kidney sections (**1**–**4**) show: (**1**) (×2800) control rat; (**2**) rat treated with 1/10 MAD showing (×4400) apoptosis of tubular cells (arrows); and (**3**) (×2800), (**4**) (×3500)) rats treated with 1/5 MAD and 1/3 MAD, respectively, showing necrosis of tubular cells (arrows).

The results of histopathological analysis, performed using light microscopy and transmission electronic microscopy (TEM) techniques, of rat kidney tissues after 14-day treatment with 1/10, 1/5, and 1/3 MAD of mono-WD POM are presented in Figure 6. The photographs of the HE-stained kidney tissues show that there is no difference between the kidney tissues from the untreated group (control) (a,b) and rats treated with 1/10 MAD (c,d) and 1/5 MAD (e,f), whereas a necrosis of tubular cells (arrowhead) and glomerular sclerosis (arrows) were observed in the 1/3 MAD group (g,h). TEM analysis confirmed the necrosis of tubular cells after 1/3 MAD administration (4) and additionally revealed a similar effect in 1/5 MAD (3) as well as an apoptosis of tubular cells in the 1/10 MAD group (2).

#### 2.4.2. Histopathological Evaluation of Mono-WD POM—Induced Hepatotoxicity

**Figure 7:**
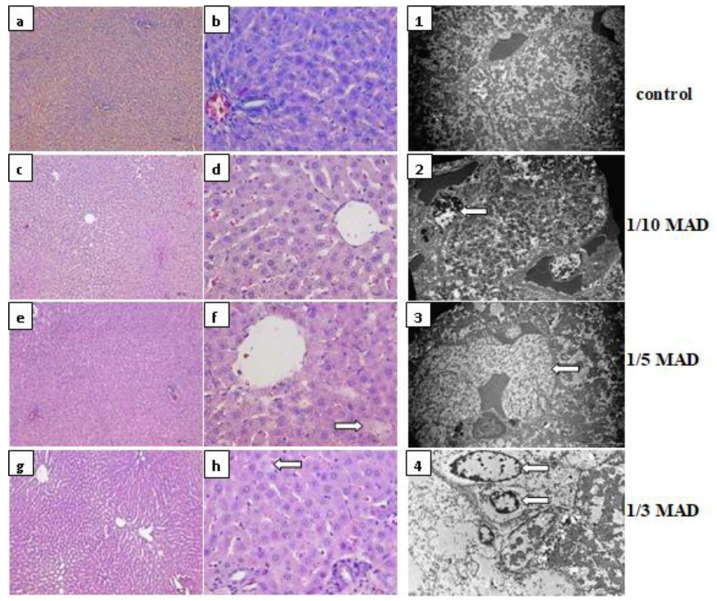
Histopathological evaluation of mono-WD POM-induced hepatotoxicity. Photographs of HE-stained rat liver sections (**a**–**h**); image was captured under a light microscope with ×10 (**a**,**c**,**e**,**g**) and ×40 (**b**,**d**,**f**,**h**) magnifications. No differences were observed between the liver tissue from the control group (**a**,**b**) and rat treated with 1/10 MAD (**c**,**d**), whereas tissue from rats treated with 1/5 MAD (**e**,**f**) showed discrete necrosis (arrow), and 1/3 MAD (**g**,**h**) showed dilatation of sinusoidal spaces and discrete necrosis (arrow). TEM micrographs of liver sections (**1**–**4**) show: (**1**) (×2200) control rat; (**2**) (×2800) rat treated with 1/10 MAD showing apoptosis of endothelial cells (arrow); and (**3**) (×2200), (**4**) (×3500)) rats treated with 1/5 MAD and 1/3 MAD, respectively, showing necrosis of hepatocytes (arrows).

Photographs of rat liver sections excised from the untreated (control) and treated rats (1/10, 1/5, and 1/3 MAD of mono-WD POM), captured under a light microscope and TEM, are shown in Figure 7. Photomicrographs of HE-stained liver tissues demonstrated that the administration of 1/5 (e,f) and 1/3 MAD (g,h) induced discrete necrosis. Moreover, a necrosis of hepatocytes was also visible under TEM (3,4) for these experimental groups. On the other hand, normal tissue morphology (as in the control group (a,b)) was seen in the liver tissues of 1/10 MAD-treated rats (c,d) using light microscopy, whereas apoptosis of endothelial cells was detected in TEM micrographs obtained for this experimental group (2).

#### 2.4.3. Histopathological Evaluation of Mono-WD POM—Induced Lung Toxicity

**Figure 8:**
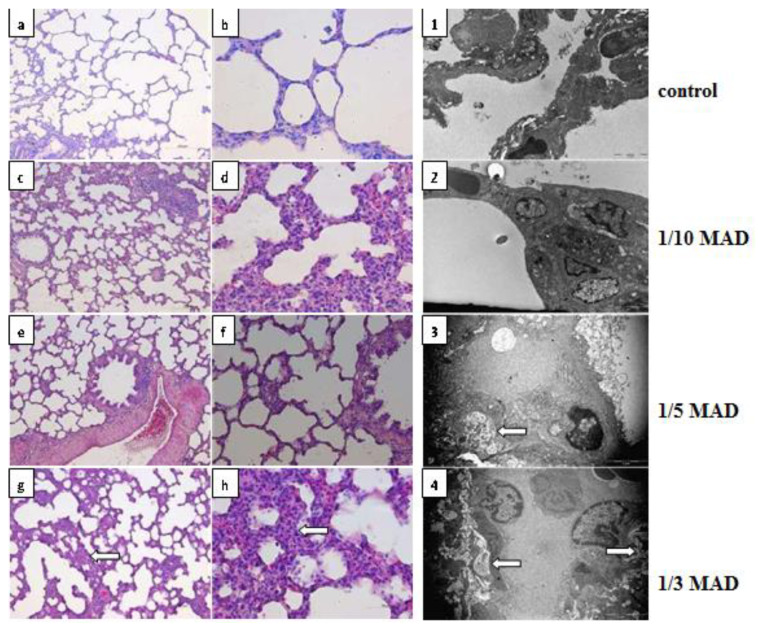
Histopathological evaluation of mono-WD POM-induced lung toxicity. Photographs of HE-stained rat lung sections (**a**–**h**); image was captured under a light microscope with ×10 (**a**,**c**,**e**,**g**), ×20 (**f**), and ×40 (**b**,**d**,**h**) magnifications. No differences were observed between the lung tissues from the control group (**a**,**b**) and rats treated with 1/10 MAD (**c**,**d**) and 1/5 MAD (**e**,**f**), whereas tissue from rats treated with 1/3 MAD (g, h) showed a thickening of interstitial spaces (arrows). TEM micrographs of lung sections (**1**–**4**) show: (**1**) (×2800) control rat; (**2**) (×3500) rat treated with 1/10 MAD showing normal lung tissue; and (**3**) (×8900), (**4**) (×4400)) rats treated with 1/5 MAD and 1/3 MAD, showing fibrosis and prominent fibrosis of lung interstitium (arrows), respectively.

TEM micrographs and photographs of HE-stained lung tissues analyzed after rat treatment with 1/10, 1/5, and 1/3 MAD of mono-WD POM are presented in Figure 8. The results of both light microscopy and TEM analysis showed that 1/10 treatment did not induce any histological changes in the lung sections (c,d,2) compared with the control (untreated) group (a,b,1). However, a thickening of the interstitial spaces was observed in HE-stained lung sections excised from 1/3 MAD-treated rats (g,h), whereas TEM micrographs detected a fibrosis and a prominent fibrosis of lung interstitium in both 1/5 MAD (3) and 1/3 MAD (4) experimental groups.

#### 2.4.4. Histopathological Evaluation of Mono-WD POM—Induced Cardiotoxicity

**Figure 9:**
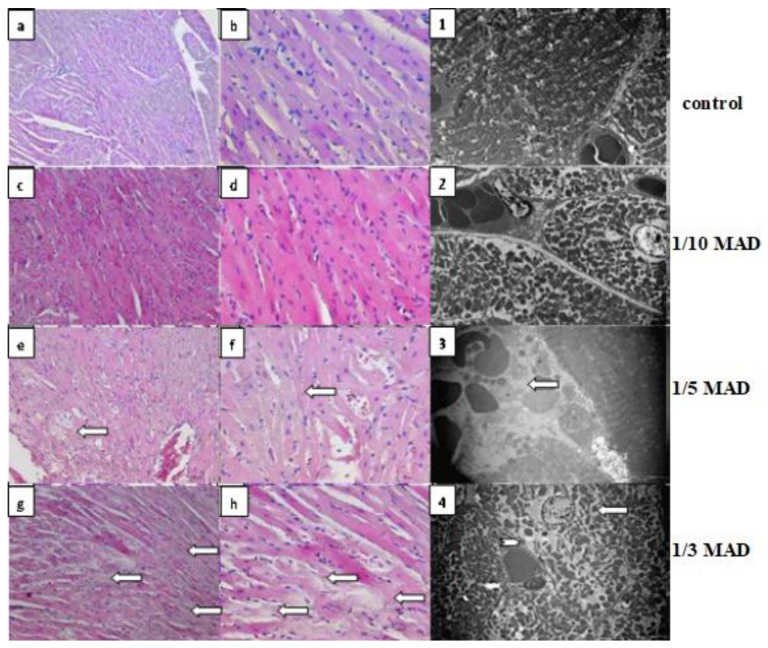
Histopathological evaluation of mono-WD POM-induced cardiotoxicity. Photographs of HE-stained rat heart sections (**a**–**h**); image was captured under a light microscope with ×10 (**a**), ×20 (**c**,**e**,**g**), and ×40 (**b**,**d**,**f**,**h**) magnifications. No differences were observed between the heart tissues from the control group (a, b) and rats treated with 1/10 MAD (**c**,**d**), whereas tissues from rat treated with 1/5 MAD (**e**,**f**) and 1/3 MAD (**g**,**h**) showed a loss of striation in cardiac muscle cells (arrows). TEM micrographs of heart sections (**1**–**4**) show: (**1**) (×2800) control rat; (**2**) (×2800) rat treated with 1/10 MAD showing normal heart tissue; and (**3**) (×3300) rats treated with 1/5 MAD showing an interstitial bleeding (arrow); (**4**) (×2800) rats treated with 1/3 MAD showing necrosis of cardiac muscle cell (arrow) and necrosis of endothelial cell (arrowhead).

The results of light microscopy and TEM analysis obtained for rat heart tissues from the control and treated groups (the application of 1/10, 1/5, and 1/3 MAD of mono-WD POM) are shown in Figure 9. No difference was obtained between the heart tissues from the control group (a,b,1) and rats treated with 1/10 MAD (c,d,2), indicating that this lowest administrated dose did not induce cardiotoxicity in the experimental rats. On the contrary, photomicrographs of HE-stained heart sections revealed a loss of striation in cardiac muscle cells (arrows) in rats treated with 1/5 MAD (e,f) and 1/3 MAD (g,h). Moreover, interstitial bleeding (arrow) was observed in TEM micrographs of heart sections in rats treated with 1/5 MAD (3), whereas necrosis of cardiac muscle and endothelial cells (arrow) was detected in 1/3 MAD-treated experimental animals (4).

### 2.5. Biodistribution Study In Vivo

**Figure 10:**
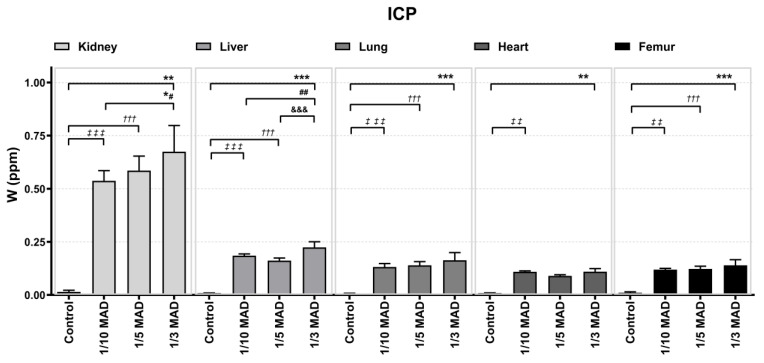
Tissue distribution of mono-WD POM in *Wistar* rats two weeks post-intravenously applied 1/10, 1/5, and 1/3 MAD, expressed as tungsten concentration (mean ± SD) in kidney, liver, lung, heart, and femur tissues. The difference between control and treated groups was presented as: * for 1/3 MAD group, † for 1/5 MAD group, and ‡ for 1/10 MAD group. The difference between 1/3 MAD and 1/5 MAD was presented as &, and between 1/3 MAD and 1/10 MAD as #. The significant values: *** *p* < 0.001, ** *p* < 0.01, ††† *p* < 0.001, ‡‡‡ *p* < 0.001, ‡‡ *p* < 0.01, &&& *p* < 0.001, ## *p* < 0.01, # *p* < 0.05.

To investigate the in vivo biodistribution of mono-WD POM in animals subjected to 1/10, 1/5, and 1/3 of MAD (960 mg/kg), the tungsten content in kidney, liver, lung, heart, and femur tissues was measured using ICP-OES, two weeks post-intravenous administration (Figure 10). As expected, a gradual increase in the given dose of mono-WD POM resulted in a corresponding increase in tungsten concentration in all investigated tissues. The highest tungsten concentrations (ppm) were detected in the kidney samples (1/3 MAD—0.67 ± 0.12; 1/5 MAD—0.59 ± 0.07; 1/10 MAD—0.54 ± 0.05). Approximately three times lower tungsten concentrations were obtained in the liver tissue (1/3 MAD—0.22 ± 0.03; 1/5 MAD—0.16 ± 0.01; 1/10 MAD—0.18 ± 0.01). Then, the lungs were the third most deposited organ with tungsten (1/3 MAD—0.16 ± 0.04; 1/5 MAD—0.14 ± 0.02; 1/10 MAD—0.13 ± 0.02). Finally, similar tungsten concentrations were detected in the heart (1/3 MAD—0.11 ± 0.01; 1/5 MAD—0.8 ± 0.01; 1/10 MAD—0.11 ± 0.00) and femur (1/3 MAD—0.14 ± 0.03; 1/5 MAD—0.12 ± 0.01; 1/10 MAD—0.12 ± 0.01).

## 3. Discussion

This study builds on our previous research, exploring the WD polyoxotungstate nanocluster family as effective CT contrast agents with the aim to identify the compound with the most favorable toxicological profile [9].

In the current work, in vitro CT studies (Figure 1) revealed significantly better contrast performances of mono-WD POM compared to the Keggine structure WSiA, differing in shape, size, charge density, and chemical composition. Thus, the ability of a molecule to attenuate X-rays could be associated with these parameters as well, not only with the presence of tungsten atoms. Moreover, higher X-ray attenuation values were obtained for mono-WD POM respective to its structural analog WD-POM [9], which is formed by the removal of one WO unit from its parent compound. Since mono-WD POM contains fewer W atoms per nanocluster (17 W and 18 W for mono-WD POM and WD-POM, respectively), the same W concentration is reached with a higher number of mono-WD POM molecules, which might be the reason for the observed stronger X-ray attenuation. Furthermore, the studied W-containing mono-WD POM showed superior contrast properties in vitro to the standard CT contrast, I-based iohexol (Omnipaque^TM^), containing three I atoms, which is in line with our previous study on parent WD-POM [9] and the literature regarding the comparison of tungsten-based nanoparticles and commercial iodine contrast agents [20,21]. From a toxicity point of view, the key advantage of mono-WD POM is that it contains a higher quantity of W atoms (17 W) per molecule compared to the quantity of I atoms per iohexol molecule (3 I) and thus can induce a higher X-ray attenuation and better contrast in CT imaging with a lower number of moles per kg body weight.

In this study, administered mono-WD POM doses (320, 192, and 96 mg/kg) corresponding to W doses of 1.107, 0.664, and 0.332 (in mmol W/kg) were significantly higher (up to 70 times) than the dose of 0.015 mmol W/kg that is usually administered for in vivo CT imaging [20,21]. Therefore, more severe toxic effects than those observed in this study are not expected if mono-WD POM is applied in effective doses for clinical CT imaging.

During the study, no death of the experimental animals was registered. This finding is different from the results of our previous study in which parent Wells–Dawson polyoxotungstate (WD-POM) showed an unexpectedly high mortality rate after intravenous administration [9]. Comparing the applied doses of the mono-WD POM studied here with its parent analog [9], it is obvious that the maximum dose of mono-WD POM applied intravenously, 320 mg/kg (1/3 MAD), did not induce the death of any treated rat (100% survival rate, 5/5), whereas for the lower dose (1.6 times) of parent WD-POM (200 mg/kg, 1/10 MTD), the survival rate was 80% (4/5). Moreover, the higher doses of parent WD-POM, 400 and 666.7 mg/kg, resulted in a significant increase in mortality rate (the survival rates of 40 and 0%, respectively). These results suggest that mono-WD POM has a better safety profile and is safer for intravenous use. Furthermore, lack of mortality and no observed physical changes after the oral application of MAD (960 mg/kg) might be associated with a low absorption rate of mono-WD POM due to its large molecular weight. Even if mono-WD POM could be absorbed, the efflux pumps or the first-pass metabolism effect could decrease its bioavailability [22]. The low bioavailability of POM-based compounds was previously shown in a study that investigated the pharmacokinetic profile of Cs_2_K_4_Na[SiW_9_Nb_3_O_40_](POM-based compound) in rats [23]. Additionally, in our previous studies that examined the oral toxicity of polyoxotungstate-based compounds, only mild toxic effects were found, additionally confirming that these compounds probably have low bioavailability [16,19]. The low POM bioavailability was additionally corroborated by the high value of MTD (2000 mg/kg) obtained for parent WD-POM [9], which is a structural analogue of the studied mono-WD POM. The low bioavailability of POM-based compounds and consequently low toxicity after *per os* application suggest that they could be considered promising candidates for the development of a new oral CT contrast agent. Moreover, the excellent survival rate found after intravenous application indicates that mono-WD POM could also be further developed as a new intravenous CT agent.

The results of blood pH values, which were below the normal range, indicate that animals in both control and all three treated groups (1/10, 1/5, and 1/3 MAD of lacunary WD POM) were in a state of acidosis. Although there was no significant difference in the pH values between the groups, the difference was found in the values of pCO_2_, HCO_3_^−^, base excess of blood, and anion gap (Table 1). The pCO_2_ values were above the normal range and higher in all treated groups relative to the control group (within the normal range). This suggests that the respiratory system was trying to compensate for the acidosis in the control group, whereas in the treated groups, it failed to do so [24]. The pCO_2_ values in the control group were in the normal range, which implies that low pH in this group was a consequence of metabolic acidosis [24,25]. Moreover, the metabolic acidosis in the control group was confirmed with the values of base excess of blood that were below the lower limit of the normal range [24,25]. The statistically higher values of anion gap in the control group than in the 1/3 and 1/5 MAD groups additionally confirmed the development of metabolic acidosis in the control group [24,25]. The results of the blood gas analysis indicate that animals in the control group developed metabolic acidosis. Since anesthetics are known to be associated with metabolic acidosis due to an increase in lactate level [26], the most probable cause of metabolic acidosis in the control group was anesthesia. On the contrary, in the treated groups, the low values of pH together with high values of pCO_2_, as well as the values of base excess of blood and anion gap (Table 1), indicate the presence of mixed metabolic and respiratory acidosis. The significantly higher HCO_3_^−^ values in the treated groups point out that respiratory acidosis persists for a longer period [24,25]. All of this suggests that the metabolic acidosis observed both in the treated groups and in the control group is a consequence of anesthesia, whereas respiratory acidosis is a consequence of mono-WD POM-induced toxic effects on the lung. The concentration-dependent effect in blood gas analysis was noted in the treated groups, with the most pronounced effect in animals that received the highest concentration (1/3 MAD) of mono-WD POM.

CO-oximetry is used for the diagnosis of intoxication that is manifested with pulmonary symptoms. Oxyhemoglobin and methemoglobin levels were significantly lower, whereas deoxyhemoglobin levels were higher in all treated groups (Table 2). The oxyhemoglobin level was below the lower limit of the normal range, methemoglobin was in the normal range, and deoxyhemoglobin was significantly above the normal range (about six times) in groups receiving mono-WD POM. The fact that oxyhemoglobin is a form of hemoglobin with oxygen and deoxyhemoglobin is a form of hemoglobin without oxygen indicates that the animals in the treated groups were in hypoxia and that the oxygenation of blood in the lungs was poor [27]. An additional confirmation of poor blood oxygenation is the low values of oxygen saturation and oxygen content of hemoglobin, which were below the lower limit of the normal range for all three treated groups of experimental animals. Accordingly, the results of CO-oxymetry analysis indicate that the intravenous application of mono-WD POM in the applied doses could result in lung damage and respiratory acidosis. This assumption is in accordance with our previous study [9], as well as with literature reports on tungsten-associated lung pathologies [28,29].

The glucose and lactate levels (Figure 2 and Figure 3) were above the upper limit of the normal range in both control and all three treated groups. Significantly higher values of glucose, relative to the control group, were detected only in the 1/3 MAD group, whereas the lactate levels were not significantly different between the experimental groups. The elevation of glucose levels is most likely a consequence of surgical intervention and anesthesia. The surgical intervention, performed to expose organs and collect blood samples, triggered a stress induced-hyperglycemia, which is a common response to this type of intervention [30,31,32]. Similar results were obtained in our previous study that followed the same protocol [9]. In this study, the rise of glucose levels, as a consequence of anesthesia and surgical intervention, was also recorded in both control and WD-POM treated groups [9]. The significantly higher glucose values in the 1/3 MAD group point out that this POM-based compound interferes in some way with the organism’s response to stress. The higher lactate levels were also a result of stress, which initially increased glucose levels and accelerated metabolism, causing a rise in lactate levels. Furthermore, the rise in lactate level is common during anesthesia and surgical interventions [33,34,35] and explains isolated metabolic acidosis in the control group. It is also an explanation for a metabolic part of mixed respiratory and metabolic acidosis in all three treated groups, meaning that mono-WD POM induced only respiratory acidosis. Respiratory acidosis, developed as a consequence of the intravenous administration of POM-based compound, was also found in our previous study [9], additionally confirming the toxic effect of WD polyoxotungstates on the lungs.

The concentration of electrolytes measured in this study (Table 3) indicates possible kidney problems in mono-WD POM-treated animals, mainly due to overall high (above the upper limit of the normal range) Mg^2+^ levels that were significantly different only when the control group was compared to 1/3 MAD group. Ca^2+^ level was also significantly higher in the 1/3 MAD group, but in the other groups, Ca^2+^ values were within the normal range. Hypermagnesemia is commonly seen in kidney disease and is a consequence of decreased glomerular filtration rates [36]. Elevated calcium levels are common in acute kidney disease (AKD). They are usually the reason for AKD development, not a consequence of AKD [37]. Hypercalcemia occurs when there is a higher rate of absorption from the intestine or when the bones release more calcium into the blood [38]. Most probably in our case, mono-WD POM affected the bones and increased calcium release.

Considering the role of the kidney in the excretion of different compounds from the organism, as well as the fact that the use of various drugs and xenobiotics can damage the kidney and affect its function, functional and histological analysis was performed to evaluate the nephrotoxic potential of mono-WD POM. Creatinine values (Figure 4), measured from the blood two weeks after the intravenous administration of saline and mono-WD POM, were in the physiological range for all experimental groups except for 1/3 MAD, with no statistical difference between the groups. On the other hand, BUN levels (Figure 5) were in the physiological range only in the control group. A dose-dependent increase in BUN concentrations, as well as a significant difference in BUN values, was observed when comparing the control group with the 1/3 and 1/5 MAD groups.

Since a rise in creatinine and BUN levels is commonly seen in acute kidney injury (AKI) [39], based on the obtained results, it can be assumed that mono-WD POM caused AKI. Nevertheless, the increase in creatinine can be postponed, especially in the early stages. This is because serum creatinine level is also dependent on non-renal factors [39]. In our study, the most prominent effects were observed in animals treated with 1/3 MAD of mono-WD POM in which both creatinine and BUN levels were high, which indicates that the degree of AKI was dose-dependent. Since AKI develops most commonly as a consequence of acute tubular necrosis [40], the development of acute tubular necrosis in animals treated with mono-WD POM might be an explanation for the development of AKI. Additional confirmation for this hypothesis is the detected hypermagnesemia, which is a common phenomenon in tubular necrosis [41]. These findings can be ascribed to the highest deposition of tungsten in the kidneys (Figure 10) and the necrosis of tubular cells confirmed by histopathological analysis of kidney tissue (Figure 6).

The results of the biodistribution study (Figure 10) are in agreement with the highest tungsten quantity found in kidneys as the main excretory organ, as it was also reported for the parent compound (WD-POM) of mono-WD POM [9] and previously published data for polyoxotungstate, Cs_2_K_4_Na[SiW_9_Nb_3_O_40_] [23]. Approximately three times less levels of tungsten detected in the rat liver samples (0.2242 *vs.* 0.6744 ppm for 1/3 MAD) (Figure 10) are in line with the reported tissue distribution of the parent molecule (WD-POM) and consequently induced morphological irregularities in liver sections (Figure 7). Overall, a dose-response tendency of tungsten deposition was observed in all investigated tissues and corresponded to morphological changes revealed by light microscopy and TEM. For example, no histological changes, fibrosis, and prominent fibrosis of the lung tissue were observed for 1/10, 1/5, and 1/3 MAD of mono-WD POM, respectively (Figure 8).

Gadolinium-based contrast agents clinically approved in the EU and USA were reported to exhibit three types of biodistribution: extracellular with renal or mixed renal/hepatobiliary elimination and intravascular or blood pool agents with renal elimination after intravenous administration. These hydrophilic, water-soluble metal complexes can quickly be distributed to the extracellular space and be eliminated mainly via the kidneys [42,43,44]. In accordance, mono-WD POM, as a metal-containing cluster soluble in water with the highest accumulation in kidney tissue (Figure 10), is expected to belong to extracellular fluid agents that are cleared via the kidneys by glomerular filtration and were proven as very safe after an intravenous administration at high doses [44]. Furthermore, literature data on gadolinium deposition in rats and mice after the intravenous injection of the approved gadolinium-based contrasts and “free gadolinium” (gadolinium acetate/chloride) revealed remarkable gadolinium levels in the liver and bone as the main deposition organs [45,46,47]. The results of this tissue distribution study (Figure 10) also detected statistically significant tungsten concentrations in the liver and femur of *Wistar* rats two weeks post-intravenously applied 1/10, 1/5, and 1/3 MAD of mono-WD POM. Similarly, the biodistribution profile of iohexol, the standard iodine-based contrast agent for CT, revealed its existence in mice’s hearts, livers, and kidneys for seven days after intravenous application [48].

In summary, mono-WD POM demonstrated superior contrast performance compared to its parent WD-POM, Keggine structure WSiA, and the conventionally used CT contrast agent, iohexol. All administrated doses of mono-WD POM did not induce mortality of the treated animals, indicating a better safety profile in comparison with the parent WD-POM. The dose-dependent side effects were confirmed by biochemical and histological analysis, whereas the most prominent changes were found in kidney tissue in which the highest tungsten amount was accumulated. The observed biochemical changes, particularly the rise in Mg^2+^ and BUN levels in the 1/3 MAD group, confirmed metabolic acidosis and kidney function alterations. Accordingly, mono-WD POM merits further in vivo research on imaging efficacy and possible toxicity, which may facilitate the development of inexpensive, safe, and innovative CT contrast agents for clinical application. Finally, future research directions could include the latest potential strategies like deep learning to improve novel contrast agents’ effectiveness and potential innovations in imaging and biomaterial technologies [49,50,51].

## 4. Materials and Methods

### 4.1. Chemicals

Monolacunary Wells-Dawson POM, α_2_-K_10_P_2_W_17_O_61_^.^20H_2_O (mono-WD POM) was synthesized according to a previously described procedure [52] and dissolved in a saline solution for further experiments. The investigated α_2_-K_10_P_2_W_17_O_61_·20H_2_O is directly obtained by the removal of one WO octahedron from the cap position of the α–[P_2_W_18_O_62_]^6−^ structure. The structure of the synthesized mono-WD POM was validated by ^31^P NMR spectroscopy, showing characteristic peaks at δ = −7.2 and −14.4 ppm, belonging to two non-equivalent phosphorus atoms in the lacunary structure [53]. 12-tungstosilicic acid, H_4_SiW_12_O_40_ (WSiA) was purchased from Sigma–Aldrich, Munich, Germany.

A stock solution of mono-WD POM (64 mg/mL) was prepared by heating at 50 °C and intensive stirring. The stock solution was diluted with saline up to particular concentrations in order to achieve the desired application doses: 1/10 MAD (96 mg/kg), 1/5 MAD (192 mg/kg), and 1/3 MAD (320 mg/kg). The animals were treated immediately after the solutions were prepared.

### 4.2. In Vitro CT Imaging

The in vitro measurement of X-ray attenuation was performed in plastic test tubes using a clinical CT GE Medical Systems Revolution Evo 128-Detector CT scanner (the X-ray source current—400 mA, the X-ray source voltage—80 kV). The phantom images were acquired for two polyoxotungstates differing in shape and structure: Keggin structure 12-tungstosilicic acid, H_4_SiW_12_O_40_ (WSiA) and monolacunary Wells–Dawson POM, α_2_-K_10_P_2_W_17_O_61_^.^20H_2_O (mono-WD POM). The selected concentrations of the tested solutions were (in mM): 3.125, 6.25, 12.5, 25, 50, and 100. Water was used as a reference for Hounsfield units (HU) of 0.0.

### 4.3. Ethical Approval

The ethical approval for methodological procedures performed in this study was given by the Faculty of Medicine of the University of Belgrade Ethical Commission for Experimental Animal Welfare Protection, (N°6447/1-2020). All procedures performed in this study followed Guidelines from the European Convention for the Protection of Vertebrate Animals Used for Experimental and Other Scientific Purposes. Good Laboratory practice was fully applied as well. The ARRIVE (Animal Research: Reporting of In Vivo Experiments) guidelines 2.0 were used for reporting in this study [54].

### 4.4. Experimental Animals

Healthy, male, 5-week-old *Wistar albino* rats weighing 180–220 g were selected for these experiments. Rats are the preferred species for conducting toxicological studies in accordance with the guidelines outlined by the Organization for Economic Cooperation and Development (OECD) [55,56] Moreover, previous research has demonstrated the efficacy and reliability of this model [9,57]. The selected animals were housed in polycarbonate cages (3–4 animals per one cage). The husbandry conditions were standard for this type of experiment. More precisely, the animals had *ad libitum* access to standard rodent food and tap water. A temperature of 23 ± 2 °C, humidity of 55 ± 10%, and a 12/12 dark/light cycle were maintained during the whole study period.

### 4.5. Experimental Design

Since the in vivo toxicity of mono-WD POM was tested for the first time in this study, the recommended protocol [55] was applied for the selection of doses and the follow-up period. First, experimental rats received single doses of 5, 50, 300, and 960 mg/kg *per os*, and their general habits were monitored. The animals were observed individually during the period of 14 days. A dose of 2000 mg/kg, which is recommended by the protocol, was not applied because the maximum solubility of the studied mono-WD POM was 64 mg/mL, which allowed a maximum dose of 960 mg/kg to be administered. Considering the limited solubility of the studied mono-WD POM (maximum solubility—64 mg/mL), a maximum-administered dose (MAD) of 960 mg/kg was used for an acute oral toxicity evaluation [58] instead of the maximum-tolerated dose (MTD) [9,55,56]. Four experimental groups were created for this study: the control group (received saline intravenously) and three treated groups created based on the proportion of the intravenously administered MAD dose of mono-WD POM [9,57,59]. The first treated group received 1/3 of MAD (320 mg/kg), the second group received 1/5 of MAD (192 mg/kg), and the third group received 1/10 of MAD (96 mg/kg).

**Scheme 2:**
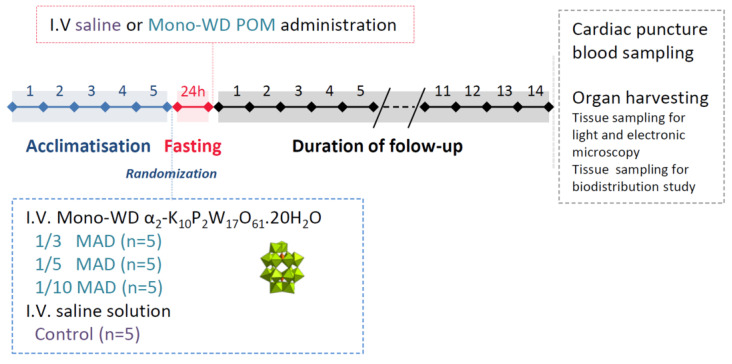
Experimental design for in vivo acute toxicity evaluation of monolacunary Wells–Dawson polyoxotungstate (mono-WD POM).

At the beginning of the experiment, the animals were brought from a central housing facility and were given five days to acclimate to laboratory housing conditions. Twenty-four hours before saline or mono-WD POM application, food was withdrawn while leaving free access to tap water. Both saline and mono-WD POM were administered into the lateral rat tail vein in a single dose that did not exceed 1 mL. An intravenous approach was used because mono-WD POM is expected to be used as an intravenous contrast agent. Instantly, after the administration of saline/mono-WD POM, the animals were given access to food. For the next 14 days, after saline/mono-WD POM administration, animals were twice daily observed for any changes in action that deviated from their usual behavior, physical changes, or for the presence of visible toxicity symptoms. The detailed study design is depicted in Scheme 2.

Before the study started, the exact number of animals needed was calculated to follow the 3R rule (replacement, reduction, and refinement of experimental animals). For the calculation of the sample size, power analysis was performed, and the obtained results were checked with the resource equation method [60]. In the power analysis, the effect size and standard deviation were taken from an initial study [16], the type I error was set at 5%, power was 80%, a two-tailed test was used, and an expected attrition of sample size was set at 20%. The calculated number of animals was twenty (five in each group).

To ensure that each animal has an equal probability of receiving a particular treatment (saline or the selected proportions of MAD) and to achieve an equal number of animals in each group, a randomization procedure was performed during the allocation of animals to the control and treated groups. For randomization, the random numbers were generated using the standard *= RAND ()* function in Microsoft Excel(v2401) ^®^.

### 4.6. Blood Sampling and Organ Harvesting

After a 14-day follow-up period, the animals were weighed, and the weight was used to determine the dose of anesthetic urethane. In the morning of the last day of the experimental protocol, urethane was prepared and administrated as a single dose. The anesthetic was given intraperitoneally at a dose of 125 mg per 100 g of body weight. After the induction of anesthesia and a stabilization process, which took 90 min, the depth of anesthesia was determined with a needle poking the tail and tweezers pinching the space between the toes on the back paw. The lack of response to tail poking with a needle was considered superficial anesthesia, while the cessation of pedal withdrawal reflex after pinching was considered a state of deep surgical anesthesia.

After reaching a state of surgical anesthesia, the rat’s chest and abdomen were opened to expose the animal’s organs. For arterial blood gas analysis, a heparinized syringe was used. Arterial blood was sampled by entering the needle into the exposed left heart. After blood collection, to expose the blood to heparin and prevent unvented clotting, the syringe was thoroughly mixed. Once the blood was collected, the animals were decapitated, and the following organs were harvested from each *Wistar albino* rat: kidney, liver, lung, heart, and femur. The organs harvested for light microscopy were fixed in 4% buffered formaldehyde. For electronic microscopy, the organs were fixed in 3% glutaraldehyde. The tissue samples (kidney, liver, lung, heart, and femur) were taken for the biodistribution study and kept at –80 °C. All blood samples for gas analysis and tissue samples for histological and biodistribution studies were coded. The codes were revealed after completing all analyses to enable statistical calculations.

### 4.7. Laboratory Blood Analysis

The Profile Prime Plus^®^ Critical Care Analyzer (Nova Biomedical Co., Waltham, MA, USA) was used for blood gas analysis (pH, pCO_2_, HCO_3_^−^, base excess of blood, and anion gap), CO–oximetry (total hemoglobin, oxyhemoglobin, carboxyhemoglobin, methemoglobin, deoxyhemoglobin, oxygen saturation, oxygen content of hemoglobin, and oxygen capacity of hemoglobin) analysis, and for measuring electrolytes (Na^+^, K^+^, Cl^−^, Ca^2+^, Mg^2+^, Ca^2+^ normalized to pH 7.4, and Mg^2+^ normalized to pH 7.4), namely, glucose, lactate, creatinine, and blood urea nitrogen (BUN) levels. These analyses were performed immediately after the blood was collected.

### 4.8. Histological Analysis

For histological analysis by light microscopy, tissue samples (kidney, liver, lung, and heart) were first fixed and then embedded in paraffin. Then, the tissues were cut into 5 μm-thick sections and stained with Hematoxylin and Eosin (HE). HE-stained micrographs were taken using a light microscope (Leica DMLS) with a digital camera (Leica DFC295).

TEM analysis was carried out with the tissue samples that were first fixed with 3% glutaraldehyde in cacodylate buffer. After fixation in 1% OsO_4_ and dehydration in graded alcohols, the samples were fixed in Epoxy medium (Sigma-Aldrich, 45345). Then, thin tissue sections were prepared and mounted on copper grids (Sigma–Aldrich, G4901). After staining with uranyl acetate and lead citrate, the samples were analyzed under an electron microscope (Morgagni 268D, FEI, Hillsboro, OR, USA).

### 4.9. Biodistribution Study In Vivo

0.1 g of tissue sample (kidney, liver, lung, heart, or femur), 4 mL HNO_3_, 5 mL HCl, and 2 mL HBF_4_ were added to a Teflon vessel. The pre-set ‘Easy Prep *i*wave Zeolite’ protocol was applied for digesting the samples using a MARS6 microwave (15 min ramp time to 210 °C, 15 min hold at 210 °C, 30 min cool down before opening the vessel). Then, the digested sample solutions were diluted (Milli-Q water) to 2% HNO_3_ (final volume of 10 mL).

The digested tissue samples were analyzed by inductively coupled plasma–optical emission spectroscopy (ICP-OES), using a PerkinElmer optical emission spectrometerAvio 500 instrument. A calibration curve was prepared with a series of standard tungsten solutions in the concentration range of 0.0001–50 ppm.

### 4.10. Statistical Analysis

For statistical analysis, the Graph Pad Prism (Graph Pad Software, San Diego, CA, USA) software was used. The difference between the four groups was calculated with one-way analysis of variance (ANOVA) followed by a Tukey post hoc test and was considered significant if *p* ˂ 0.05. The obtained results are expressed as mean ± standard deviation (SD). The presented *p*-values in both the text and tables of this paper indicate significance determined through one-way analysis of variance. The distinctions between the control and experimental groups (1/10 MAD, 1/5 MAD, and 1/3 MAD) reflect the outcomes of post hoc Tukey analysis and are denoted in tables and graphs by the symbols: *, †, and ‡.

## Figures and Tables

**Table 1:** The results of arterial blood gas analysis for the control group and animals treated with 1/10 MAD, 1/5 MAD, and 1/3 MAD of mono-WD POM.

Analyte	Units	Control (n = 5)	1/10 MAD (n = 5)	1/5 MAD (n = 5)	1/3 MAD (n = 5)	ANOVA *p*-Value	Normal Range
pH	/	7.16 ± 0.03	7.16 ± 0.03	7.28 ± 0.17	7.07 ± 0.12	0.0545	7.3–7.4
pCO_2_	kPa	4.97 ± 1.02	9.84 ± 2.54	9.92 ± 4.50	15.78 ± 3.57	0.0007 ***	4.66–5.99
HCO_3_^−^	mmol/L	17.52 ± 3.48	26.43 ± 5.57	29.79 ± 2.46	34.41 ± 1.77	<0.0001 ***†††‡‡	21–28
Base excess of blood	mmol/L	(−) 6.64 ± 1.54	(−) 2.85 ± 3.47	(−) 0.7 ± 1.79	1.56 ± 2.86	0.0008 ***†	−2 to +3
Anion gap	mmol/L	15.94 ± 1.88	13.60 ± 2.57	9.01 ± 3.53	5.11 ± 1.45	<0.0001 ***††	8.00–16.00

For determining the difference in values of arterial blood gas analysis between control and three treated groups, one-way ANOVA was used, followed by the Tukey post hoc test. After Tukey post hoc analysis, the difference between the control and treated groups was presented as: * for 1/3 MAD group, † for 1/5 MAD group, and ‡ for 1/10 MAD group. The significant values: *** *p* < 0.001, ††† *p* < 0.001, †† *p* < 0.01, † *p* < 0.05, ‡‡ *p* < 0.01.

**Table 2:** The results of CO-oximetry analysis obtained for the control group and animals treated with 1/10 MAD, 1/5 MAD, and 1/3 MAD of mono-WD POM.

Analyte	Units	Control (n = 5)	1/10 MAD (n = 5)	1/5 MAD (n = 5)	1/3 MAD (n = 5)	ANOVA *p*-Value	Normal Range
Total hemoglobin	g/L	164.50 ± 7.02	173 ± 10.97	173.20 ± 13.88	156.40 ± 8.96	0.0669	140–178
Oxyhemoglobin	%	97.87 ± 0.52	67.36 ± 12.78	60.04 ± 7.88	73.61 ± 13.69	0.0001 **†††‡‡	94–97
Carboxyhemoglobin	%	0.45 ± 0.04	0.43 ± 0.03	0.7 ± 0.46	0.61 ± 0.31	0.3869	0.0–1.5
Methemoglobin	%	0.82 ± 0.29	0.39 ± 0.04	0.4 ± 0.07	0.40 ± 0.06	0.0008 **††‡‡	0.0–1.5
Deoxyhemoglobin	%	0.55 ± 0.07	30.91 ± 11.68	36.92 ± 4.22	23.93 ± 10.96	<0.0001 **†††‡‡‡	0.0–5.0
Oxygen saturation	%	97.8 ± 3.82	69.50 ± 14.16	60 ± 8.68	74.63 ± 15.67	0.004 ††‡	95–98
Oxygen content of hemoglobin	vol %	21.97 ± 3.46	14.93 ± 5.04	13.38 ± 3.50	16.18 ± 3.61	0.0202 †	18–24
Oxygen capacity of hemoglobin	vol %	23.42 ± 1.85	23.89 ± 1.56	24.04 ± 1.96	21.24 ± 1.21	0.0994	18–25

For determining the difference in CO-oximetry values between control and three treated groups, one-way ANOVA was used, followed by the Tukey *post hoc test. After* Tukey post hoc analysis, the difference between control and treated groups was presented as: * for 1/3 MAD group, † for 1/5 MAD group, and ‡ for 1/10 MAD group. The significant values: ** *p* < 0.01, ††† *p* < 0.001, †† *p* < 0.01, † *p* < 0.05, ‡‡‡ *p* < 0.001, ‡‡ *p* < 0.01, ‡ *p* < 0.05.

**Table 3:** Electrolyte concentrations in arterial blood obtained for the control group and treated animals (1/10, 1/5, and 1/3 MAD of mono-WD POM).

Analyte	Units	Control (n = 5)	1/10 MAD (n = 5)	1/5 MAD (n = 5)	1/3 MAD (n = 5)	ANOVA *p* Value	Normal Range
Na^+^	mmol/L	135.8 ± 1.52	135.9 ± 3.02	137.7 ± 2.78	135.4 ± 4.12	0.6297	136–146
K^+^	mmol/L	5.42 ± 0.46	5.59 ± 0.55	5.22 ± 0.49	5.52 ± 0.26	0.6168	3.5–5.2
Cl^−^	mmol/L	103.70 ± 2.39	102.0 ± 1.39	104.10 ± 2.63	103.8 ± 3.33	0.554	98–106
Ca^2+^	mmol/L	1.23 ± 0.04	1.27± 0.04	1.29 ± 0.06	1.39 ± 0.08	0.0058 **	1.09–1.30
Mg^2+^	mmol/L	0.66 ± 0.10	0.96 ± 0.13	0.95 ± 0.16	1.32 ± 0.23	0.0001 ***	0.45–0.65
Ionized Ca^2+^ normalized to pH 7.4 (nCa)	mmol/L	1.12 ± 0.04	1.12 ± 0.03	1.22 ± 0.08	1.19 ± 0.07	0.0151 †	1.09–1.30
Ionized Mg^2+^ normalized to pH 7.4 (nMg)	mmol/L	0.61 ± 0.04	0.83 ± 0.10	0.89 ± 0.23	1.04 ± 0.13	0.002 **†	0.45–0.6

For determining the difference in electrolyte concentrations between the control and three treated groups, one-way ANOVA was used, followed by the Tukey post hoc test. After Tukey post hoc analysis, the difference between the control and treated groups was presented as: * for the 1/3 MAD group and † for the 1/5 MAD group. The significant values: *** *p* < 0.001, ** *p* < 0.01, † *p* < 0.05.

## Data Availability

The data presented in this study are available upon request from the corresponding author.
